# Early Efficacy of Type I Collagen-Based Matrix-Assisted Autologous Chondrocyte Transplantation for the Treatment of Articular Cartilage Lesions

**DOI:** 10.3389/fbioe.2021.760179

**Published:** 2021-10-28

**Authors:** Xiang Li, Shiao Li, Jiatian Qian, Yancheng Chen, Yiqin Zhou, Peiliang Fu

**Affiliations:** Department of Arthroplasty Surgery, Changzheng Hospital, Naval Medical University, Shanghai, China

**Keywords:** articular cartilage, autologous chondrocyte transplantation, collagen, lesion, hyaline

## Abstract

**Background:** Articular cartilage is a complex structure that allows for low frictional gliding and effective shock absorption. Various sports injuries and inflammatory conditions can lead to lesions in the articular cartilage, which has limited regenerative potential. Type I collagen combined with autologous chondrocytes in a three-dimensional culture were used to induce the regeneration of single-layer autologous expanded chondrocytes without chondrogenic differentiation.

**Purpose:** To assess the clinical, radiological, and histological changes following collagen-based autologous chondrocyte transplantation (MACT) for chondral knee lesions.

**Methods:** The study prospectively enrolled 20 patients with symptomatic knee chondral lesions (mean size lesion was 2.41 ± 0.43 cm^2^, range: 2.0–3.4 cm^2^) in the lateral femoral condyle and femoral groove who underwent type I collagen-based MACT between July 2017 and July 2019. knee injury and osteoarthritis outcome score (KOOS) was assessed before the procedure, and periodic clinical follow-up was conducted every 3 months for a maximum of 12 months following the procedure and at 1-year intervals thereafter. Magnetic resonance imaging (MRI) T2 mapping of repaired cartilage was also used for the quantitative analysis of regeneration. In one patient, second-look arthroscopy was performed to assess cartilage regeneration characteristics, and a portion of regenerated cartilage was harvested for histological evaluation 12 months after implantation.

**Results:** At pre-operation and at three, six, 12, and 24 months after the operation, KOOS pain, symptoms, daily life activities, sports and recreation, as well as the quality of life were significantly improved between every two time points. Hematoxylin and eosin (HE) staining indicated that the newly formed cartilage was comprised of naive chondrocytes. Safranin O-fast (S-O) green staining of the regenerated tissue revealed fibroblast-like cells surrounded by glycosaminoglycans. Immunohistochemistry (IHC) analysis indicated that collagen type II was uniformly distributed at the deep zone of articular cartilage and type I collagen mainly depositing in the superficial cartilage layer. The T2 values for repaired tissue gradually decreased, eventually approaching near-average values.

**Conclusion:** The present study demonstrated that type I collagen-based MACT is a clinically effective treatment for improving functionality and pain levels. Histological evidence confirmed hyaline cartilage induction and showed that repaired cartilage tended to emerge from the deep to the superficial layer. The quantitative MRI T2 mapping test indicated that there still was a difference between the transplanted cartilage and the surrounding hyaline cartilage. Taken together, the current method represents an efficient approach for the restoration of knee cartilage lesions.

## Introduction

Hyaline cartilage has a unique capacity to cope with pressure transformations and is thus essential for proper musculoskeletal function. Unfortunately, hyaline cartilage lesions seldom heal spontaneously owing to the avascular and aneural surroundings, which limit healing capacity (Carey et al., 2020). Patients with symptomatic lesions often experience long-standing activity-related knee pain and swelling. Several methods, including microfracture, osteochondral autograft transfer, mosaicplasty, and osteochondral allografts, have been introduced for the treatment of high-level cartilage damage in young active patients (Krill et al., 2018). However, none of the above methods can completely regenerate cartilage and restore its biological functions (Levine et al., 2009; Christensen et al., 2020; Solheim et al., 2020).

The most promising method for overcoming hyaline articular cartilage’s limited intrinsic healing potential is autologous chondrocyte implantation (ACI) (Grevenstein et al., 2021). In ACI, chondrocytes are applied to the damaged area together with a membrane, such as the tibial periosteum or a biomembrane. Alternatively, chondrocytes may be pre-seeded in a scaffold matrix. First-generation ACI involves the addition of a cultured chondrocyte solution to the lesion site with a periosteum flap. Owing to the use of an autologous periosteum flap derived from a separate tibial incision, this method can lead to a longer operation time and potential graft hypertrophy (Niemeyer et al., 2014; Krill et al., 2018; Andriolo et al., 2020). The second-generation ACI method, which relies on collagen membranes, has demonstrated greater regenerative potential (Niemeyer et al., 2014).

Nevertheless, there are increasing concerns regarding the uneven distribution of chondrocytes within the lesion and the potential risks of cell leakage, with both of these problems remaining unresolved. Since the monolayer expansion of chondrocytes hinders the reconstruction of cartilage-specific matrix, autologous cell-based cartilage therapies are limited by chondrocyte dedifferentiation (Ozturk et al., 2017). Cell-to-cell contact is known to favor chondrogenic differentiation, and three-dimensional cultures induce the redifferentiation of monolayer-developed autologous chondrocytes (Jimenez et al., 2015). Third-generation ACI, also known as matrix-associated ACI (MACI), employs a three-dimensional cell culture, which more accurately mimics the microenvironment of cell growth *in vivo* (Xu et al., 2019).

Based on preclinical studies, we described the efficacy of three-dimensional type I collagen combined with autologous chondrocytes for the treatment of cartilage lesions. We further evaluated the procedure’s efficacy based on repair tissue morphology, which was assessed via MRI and histological analyses.

## Materials and Methods

### Study Design and Patients

An observational study limited to 20 cases (eight women and 12 men) was approved by the Science and Technology Committee of Shanghai and was performed at the hospital between July 2017 and July 2019. Twenty patients with isolated full-thickness cartilage lesions of the knee (measuring more than 2 cm in diameter, mean size 2.41 ± 0.43 cm; International Cartilage Repair Society (ICRS) grade III or IV; ten groove and ten lateral femoral condyles) were enrolled. Patients were aged between 20 and 50 years (mean age 40.05 ± 8.03 years). The inclusion and exclusion criteria for patient selection are presented in Table 1.

**TABLE 1:** Inclusion and exclusion criteria.

Inclusion criteria	Exclusion criteria
①Traumatic cartilage injury (ICRS grade III or more, measuring more than 2 cm in diameter)	①The presence of an active infection or inflammatory disease such as rheumatoid arthritis
②High-grade femoral condylar cartilage lesions with or without shallow subchondral bone involvement	②Patient unable or unwilling to comply with the long rehabilitation process/activity restrictions recovery
③Recalcitrant with more than 6 months of conservative care	③Unaddressed causes of increased loading (coronal knee alignment, meniscal deficiency, ligamentous laxity, patellar maltracking)
④A size of less than 10 cm^2^ for a single lesion or 15 cm^2^ for multiple lesions with a relatively intact neighboring cartilage (ICRS grade I and II)	④Obesity (defined as BMI >35 kg/m^2^)
⑤Desire to return to sports or high-impact activities	⑤More than 50% joint space narrowing on weight-bearing radiographs
⑥Younger age, 20–50 year	

ICRS, International Cartilage Regeneration and Joint Preservation Society; BMI, Body Mass Index.

The current study was designed in accordance with the Declaration of Helsinki and was approved by the Institutional Review Board of the hospital (Approval number: 2018-024-021). Informed consent was obtained from all patients before enrollment in the study.

### Procedures

For type I collagen-based matrix-assisted autologous chondrocyte transplantation (MACT), also known as MACI, chondrocytes were isolated during the first operation, expanded for 2 weeks, and pre-seeded in a matrix *in vitro*, followed by transplantation during the second surgical procedure (Gamez et al., 2020).

**FIGURE 1:**
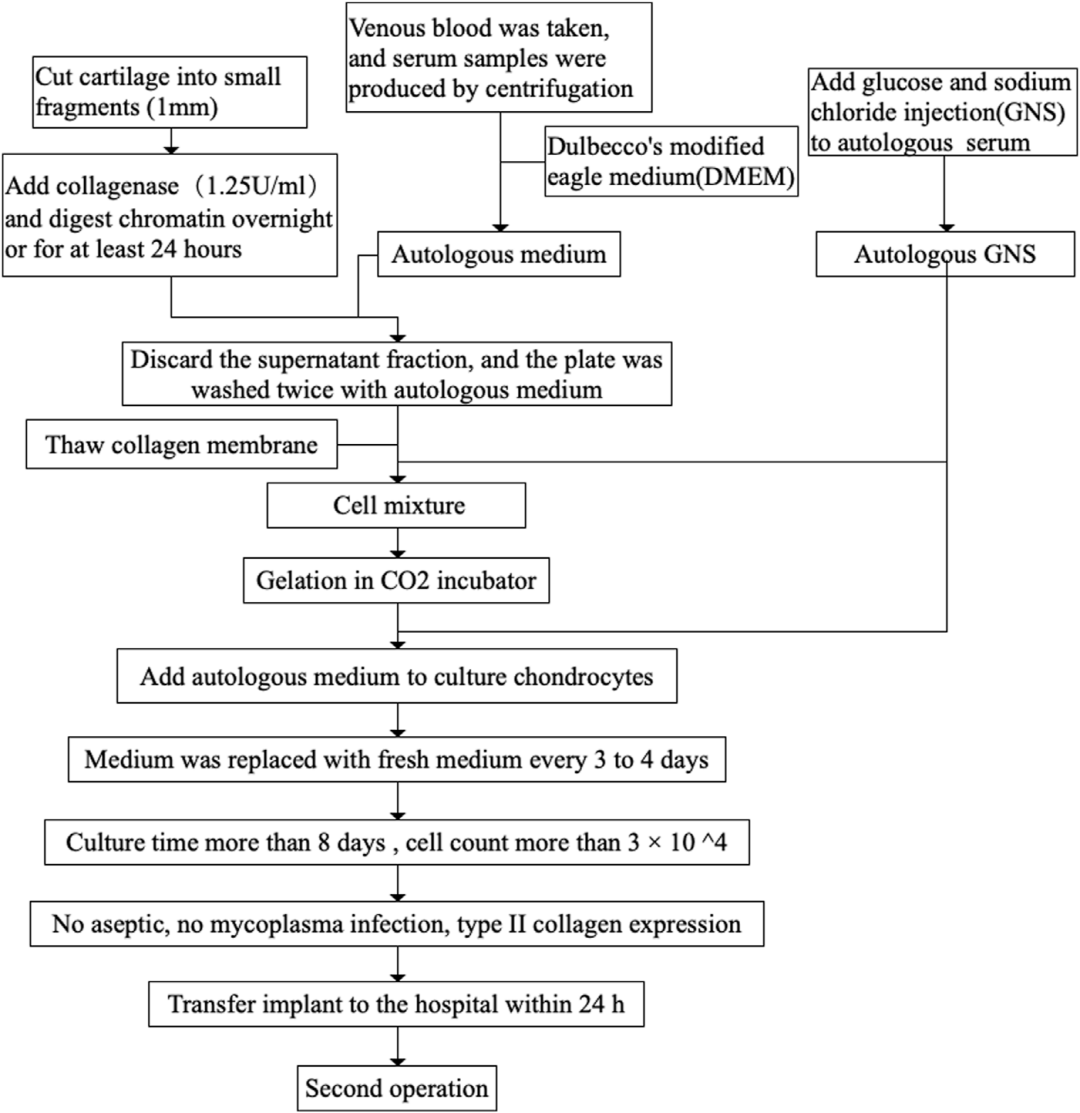
A schematic about implant for preparation. DMEM, dulbecco’s modified eagle medium; GNS, glucose and sodium chloride injection.

Approximately 150–200 ml of whole blood was collected from the patient to cultivate the implant before general anesthesia. We performed an initial arthroscopic procedure of the knee to determine the location and size of the lesion, the consistency or firmness of the adjacent articular cartilage, and the integrity of the menisci or ligaments. The cartilage biopsy specimen providing seed cells was harvested from a non-weight-bearing area of the knee via Jamshidi needle biopsy guided by an arthroscope. The sample was immediately placed in buffered serum-free medium and delivered to the laboratory with hypothermic preservation at 2–10°C. Chondrocytes were isolated from the cartilage biopsy sample through collagenase digestion for 16–24 h and obtained via density gradient centrifugation. The number of cells obtained and their viability were subsequently determined. Furthermore, the sample was preserved at 2–10°C to assess the quality over 2 months. Chondrocytes were suspended in double-buffered HEPES solution. The solution was then gently mixed with equal parts type I collagen (6 mg/ml) from rat tails in order to obtain the final concentration of type I collagen (3 mg/ml). The collagen-chondrocyte mixture was allowed to polymerize at 37°C in a humidified atmosphere. Each implant was made into two samples with a 30–40-mm diameter and two times the height (4–6 mm) of articular cartilage (2–4 mm) within the lesion area. The chondrocyte-seeded implants were cultured in autologous serum for 10–14 days (37°C, 5% CO_2_). The medium was changed every 3–4 days via adding 80 ml of fresh medium per well. Implants were then transferred to the hospital within 24 h. Before shipment, each implant had to meet the defined requirements for quality control. For instance, cellular viability had to be above 80%, as determined via Cell Counting Kit-8 (WST-8/CCK8) or flow cytometry. The number of cells had to be more than 3 × 10^4^, as determined via the grid count method. Mycoplasma detection was carried out via real-time reverse transcriptase PCR using a PCR Mycoplasma Test Kit (Applichem). Type II collagen expression was determined via DNA electrophoresis (Figure 1).

Approximately 2 weeks later, a second operation was performed. The cultured chondrocytes were reimplanted into the cartilage lesion and proliferated to produce durable load-bearing regenerative tissue over time (Brittberg, 2010; Ebert et al., 2017a). The operative procedure was performed following intravenous antibiotic administration and exsanguination of the lower extremity using a tourniquet. Medial or lateral parapatellar mini-open arthrotomy was performed to access the lesion area based on the lesion location. Chondral lesions were debrided down to the subchondral bone, and the edges of the lesion were trimmed using a sharp metal punch. The implants were prepared using a metal punch 2 mm wider than the corresponding punch used for cutting the cartilage lesion. The basal part of the cartilage lesion was coated using a Porcine Fibrin Sealant Kit (Bioseal Biotech) with a specific metal spatula as was the area around the implant before transfer into the lesion. As the hydrogel can release more than 50% of its water content, the implant was fabricated to twice the height of the lesion. It could easily be adapted to the individual shape of the cartilage lesion. In osteochondral lesions, the subchondral bone was augmented with autologous cancellous bone harvested from the tibial head. Alternatively, autologous bone cylinders from the iliac crest were used to reconstruct the subchondral plate.

### Outcome Measures

All patients were assessed on post-operative day one as well as at one, three, six, 12, and 24 months after operation based on the general condition and the knee surgical site. Pain, swelling, skin features, and infection were evaluated. Furthermore, leukocyte counts, erythrocyte sedimentation rate, and serum levels of C-reactive protein were assessed to monitor inflammatory responses on postoperative days one and three as well as at one, three, six, 12, and 24 months after operation. KOOS and magnetic resonance observation of cartilage repair tissue (MOCART) scores were determined prior to intervention as well as 3, 6, 12, and 24 months after the intervention. Repair site morphology and composition were evaluated for all patients. MRI was performed on a 3.0-T MRI scanner to obtain conventional images, and T2 mapping of repaired tissue was performed on all patients before operation as well as at 3, 6, 12, and 24 months after the operation. The T2 values for the fixed tissues were measured at each time point. A second-look arthroscopy was performed at 12 months for one patient, and a 4-mm-diameter needle biopsy was taken from the center of the repair site with patient’s informed consent. The indication for second-look arthroscopy was arthrofibrosis or meniscus lesions. This procedure was performed after MRI examination to avoid any potential influence on the MRI assessment. The morphology of chondrocytes, distribution of cells, and ECM synthesis were further studied via HE and S-O staining (Chen et al., 2014). Sections of biopsy specimens were stained with IHC to determine collagen type I and II expression.

### Rehabilitation Program

For the isolated femoral lesion, the knee joint was immobilized for 72 h in a brace locked at 10° of flexion after the operation. Knee flexion was limited to 30° during the first 3 weeks and to 60° for another 3 weeks, with partial weight-bearing for 12 weeks, consisting of 15 kg for 6 weeks and 30 kg for the following 6 weeks. Non-weight-bearing was encouraged for at least 6 weeks during the reconstruction of subchondral bone. We also recommended assisted physical therapy and bicycle training after 6 weeks as well as training for enhanced muscle formation started after 12 weeks. In all cases, continuous passive motion was recommended for 6 weeks. Physical activity, such as non-contact sports activities, swimming, and biking, were allowed after 6 months. More competitive activity loads, including soccer, track, and field athletics, were allowed 12 months after the operation.

### Statistical Analysis

We assessed changes in clinical scores and radiologically measured cartilage properties at different follow-up times using one-way ANOVA. The results obtained pre-operatively and at each follow-up were compared. Data were analyzed using SPSS23.0 and Prism 8 software with a significance threshold of *p* < 0.05.

## Results

### Adverse Events

No severe clinical adverse events were observed up to 24 months after MACT (Table 2). Joint pain, effusion, and swelling were observed in the early stages after operation, and all symptoms were ultimately improved after 8 weeks. No postoperative infections were observed up to 24 months after the procedure.

**TABLE 2:** Adverse events.

	Patients: n	Time point: wk
Joint pain	4	<4
Joint effusion	0	—
Joint swelling	2	<8
Joint stiffness	0	—
Infection at the surgical site	0	—

There are no serious adverse events among the 20 patients (i.e., new cartilage lesion in the affected knee or failure at implanted sites).

### Knee Injury and Osteoarthritis Outcome Score

**FIGURE 2:**
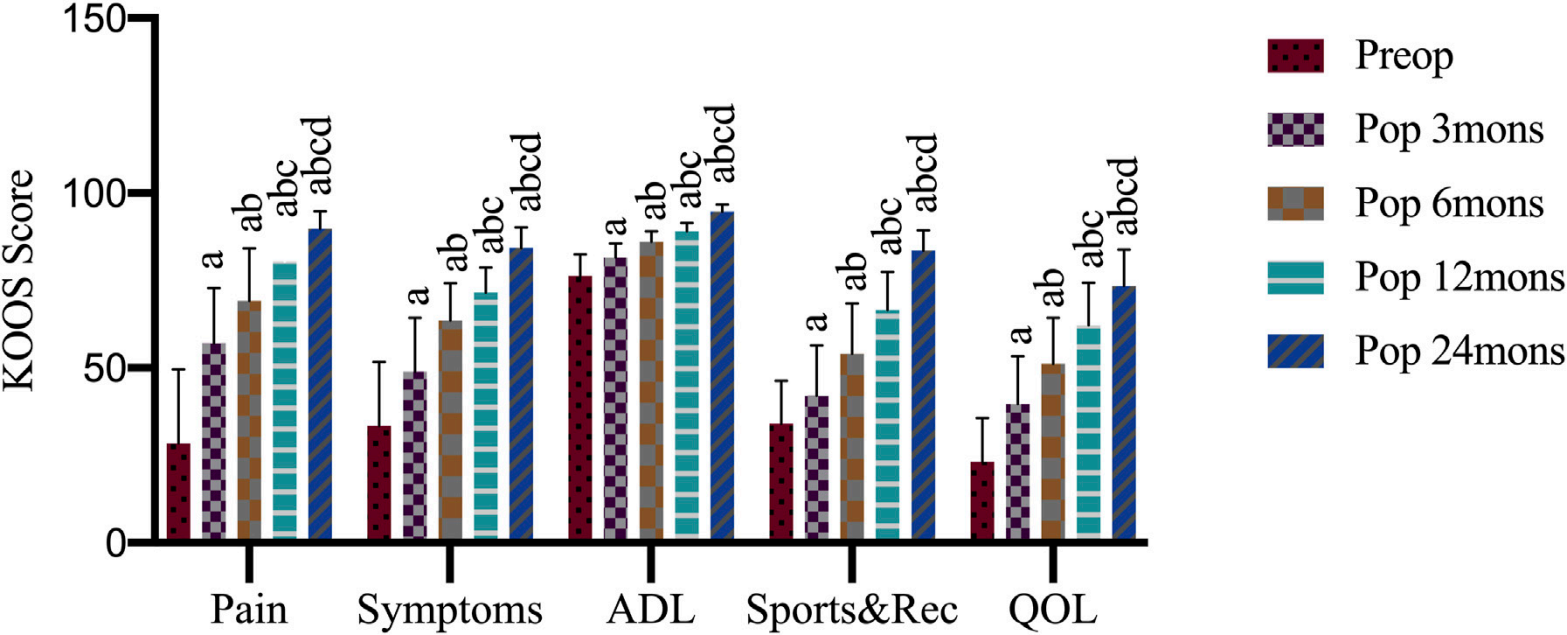
Knee injury and osteoarthritis outcome score (KOOS) outcome after operation. Preop, preoperation; Pop, postoperation; mons, months; ADL, activities of daily living; Sports and Rec, sports and recreation; QOL, quality of life; a, *p* < 0.05 (vs. preoperatively; b, *p* < 0.05 (vs. three mons); c, *p* < 0.05 (vs. six mons); d, *p* < 0.05 (vs. 12 mons).

Although somewhat variable among cases, KOOS subcategories were significantly improved after 24 months (Figure 2). At preoperation, 3, 6, 12, and 24 months after operation, the KOOS pain scores were 28.47 ± 21.09, 57.08 ± 15.73, 69.17 ± 14.97, 80.42 ± 7.67, 89.86 ± 4.96, respectively; symptom scores were 33.39 ± 18.26, 48.93 ± 15.42, 63.57 ± 10.59, 71.61 ± 7.09, 84.29 ± 5.95, respectively; daily life activity scores were 76.32 ± 6.16, 81.54 ± 3.98,86.10 ± 3.00, 89.05 ± 2.41, 94.63 ± 2.09, respectively; sports and recreation scores were 34.00 ± 12.31, 42.00 ± 14.46, 54.00 ± 14.38, 66.50 ± 10.89, 83.50 ± 5.87, respectively; quality of life scores were 23.13 ± 12.52, 39.69 ± 13.64, 51.24 ± 13.09, 62.19 ± 12.08, 73.44 ± 10.31, respectively. When compared with the preoperation values, KOOS pain (F = 56.76, *p* < 0.05), symptoms (F = 50.95, *p* < 0.001), daily life activities (F = 67.24, *p* < 0.05), sports and recreation (F = 53.80, *p* < 0.05), and quality of life (F = 49.84, *p* < 0.05) scores showed significant improvement at 3, 6, 12, and 24 months after the operation, with differences in scores between every two time points also being significant (*p* < 0.001)**.**

### Magnetic Resonance Imaging

MRI assessment indicated that cartilage lesions were filled with newly generated tissue over time. The lesion filling rate reached 100% coverage without detectable hypertrophy of the repaired tissue after 24 months for all patients. Some subchondral bone edema was observed around the implantation sites at 3 and 6 months after operation, but such abnormal signals disappeared by 24 months in all cases.

**FIGURE 3:**
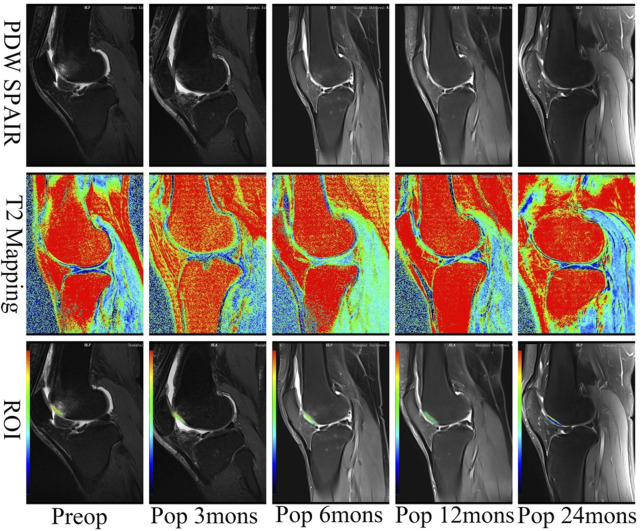
T2 mapping of cases after the operation. The chondral lesion treated with the implant developed an intensity similar to the surrounding normal cartilage over time. ROI, the region of interest. The minimum value of blue is 0 ms representing healthy cartilage; the maximum value of red is 100 ms representing damaged cartilage. PDW, proton density-weighted; SPAIR, spectral attenuated inversion recovery; mons, months.

The MRI demonstrated that the vast majority of cartilage lesions were filled and regenerated 3 months after the operation. However, integration with the surrounding cartilage was incomplete, and this significantly reduced the signal intensity of repaired tissue. Compared with results in the third month, significant progress in the integration of implanted cartilage and the surrounding cartilage was observed at 6 months after the operation, with most reaching complete integration. The signal of repaired tissue was significantly reduced, while that of subchondral bone was not. At 12 and 24 months after the operation, all indicators and the subchondral bone had returned to near-normal levels. During the recovery period, the lesion site was completely repaired and integrated with the surrounding cartilage. The signal intensity of the fixed tissue was similar to that of the surrounding tissue and subchondral bone (Figure 3). The MACT could quickly fill and repair injured cartilage, completely integrating with the surrounding cartilage in half a year. The fixed tissue reached the same signal intensity as the surrounding cartilage at 12 months after the operation.

**FIGURE 4:**
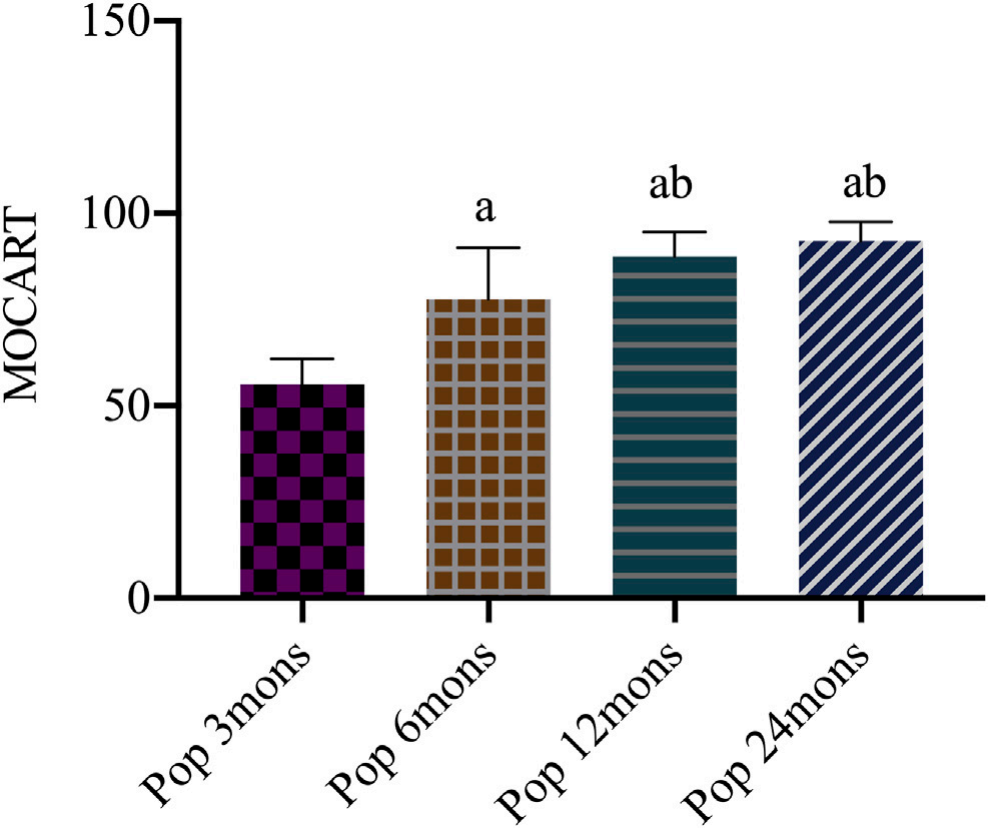
Magnetic resonance observation of cartilage repair tissue (MOCART) score after operation. Pop, postoperation; mons, months; a, *p* < 0.05 (vs. pop three mons); b, *p* < 0.05 (vs. pop six mons); c, *p* < 0.05 (vs. pop 12 mons).

The MOCART score systematically evaluates the condition of repaired tissue and surrounding normal cartilage tissue and has good reliability and repeatability. It is used for the evaluation of repaired tissue during long-term follow-up after implant. The score is based on a percentile system for the repaired cartilage as a whole with a full score of 100 points. The higher the score, the better the repaired tissue as previously described in Table 3. At different time points, the MOCART score increased significantly from 50.50 ± 6.67 at 3 months to 77.00 ± 13.42 at 6 months, 88.00 ± 6.37 at 12 months, and 92.80 ± 4.98 at 24 months. The difference between every two time points was statistically significant (F = 98.723, *p* < 0.001), except for that between 12 and 24 months (Figure 4).

**TABLE 3:** MOCART score.

Category	Item	Points
Defect fill	Subchondral bone exposed	0
	Incomplete <50%	5
	Incomplete >50%	10
	Complete	20
	Hypertrophy	15
	Subchondral bone exposed	0
	Incomplete <50%	5
	Incomplete >50%	10
Cartilage interface	Complete	15
	Demarcating border visible	10
	Defect visible <50%	5
	Defect visible >50%	0
Surface	Surface intact	10
	Surface damaged <50% of depth	5
	Surface damaged >50% of depth	0
Adhesions	Absent	5
	Yes	0
Structure	Homogeneous	5
	Inhomogeneous or cleft formation	0
Signal intensity	Normal	30
	Nearly normal	10
	Abnormal	0
Subchondral lamina	Intact	5
	Not intact	0
Subchondral bone	Intact	5
	Granulation tissue, cyst, sclerosis	0
Effusion	Absent	5
	Yes	0
Total points		100

### Magnetic Resonance Imaging T2 Mapping Values

**FIGURE 5:**
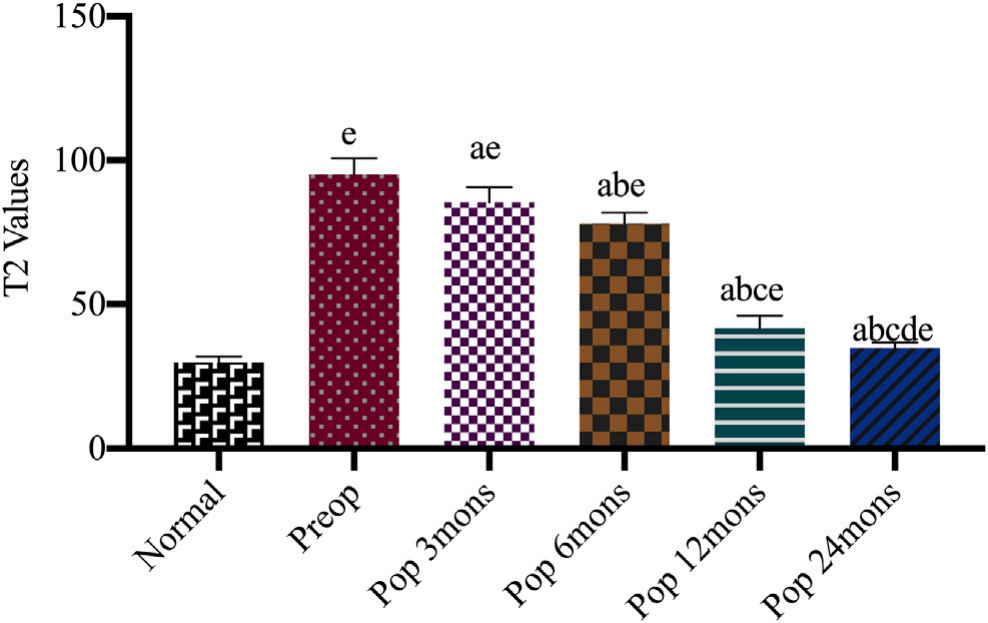
Comparison T2 values of repaired site and surrounding normal cartilage after the operation. Preop, preoperation; Posop, postoperation; mons, months; a, *p* < 0.05 (vs. preoperatively); b, *p* < 0.05 (vs. three mons); c, *p* < 0.05 (vs. six mons); d, *p* < 0.05 (vs. 12 mons); e, *p* < 0.05 (vs. normal cartilage).

T2-mapping is a multi-slice multi-echo spin-echo imaging technology with adding a spectral sequence generating a pseudo-color through the workstation post-processing software. It can quantitatively evaluate the changes of tissue to diagnose early osteochondral lesions by measuring the region of interest (ROI) for the T2 relaxation time. The minimum value of blue is 0 ms representing healthy cartilage; the maximum value of red is 100 ms representing damaged cartilage. T2 mapping revealed that treated chondral lesions achieved similar intensity to that of surrounding cartilage over time (Figure 5). We got some specific T2 values according to the pseudo-color derived from post-processing software. At different time points, the T2 values decreased significantly from 95.05 ± 5.61 before operation to 85.40 ± 5.33 at 3 months, 78.05 ± 3.80 at 6 months, 41.85 ± 4.16 at 12 months, and 34.85 ± 2.01 at 24 months. The T2 values for repaired tissue gradually decreased to approach near-average values (*p* < 0.05), and the difference between every two time points was statistically significant (F = 976.11, *p* < 0.001). Moreover, a difference was observed between the implanted cartilage and the surrounding normal hyaline cartilage (with a T2 value of approximately 29.80 ± 2.12 ms) (Figure 5).

### Second Look

**FIGURE 6:**
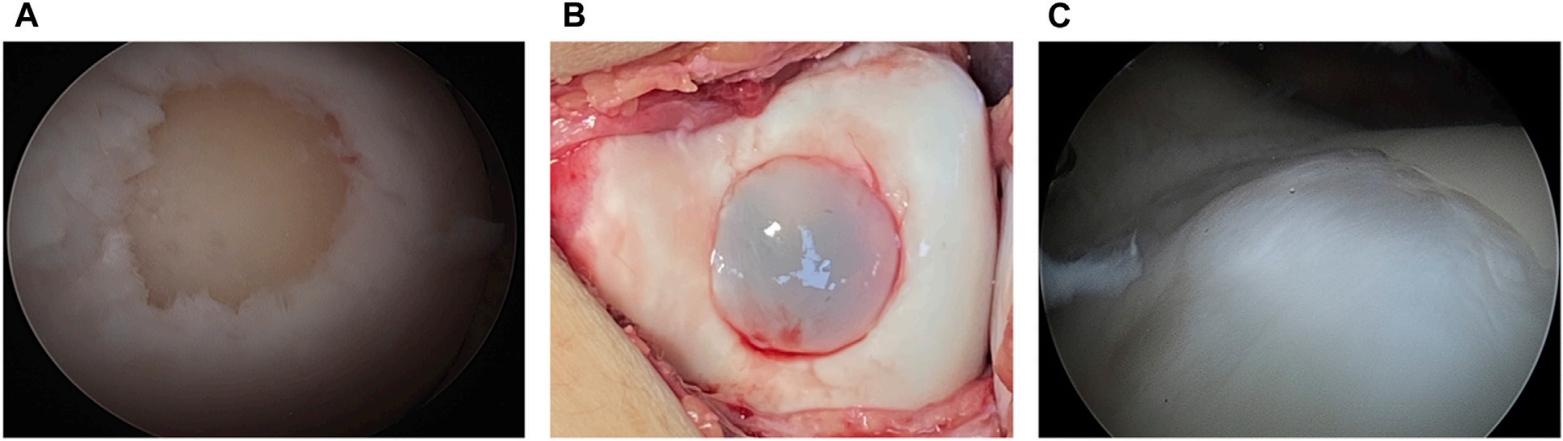
Arthroscopic findings **(A)** arthroscopic outcome before implantation **(B)** the implant is transplanted in the lesion **(C)** a second-look arthroscopy at 12 months. The lesion is covered with cartilage-like tissue after the matrix-associated autologous chondrocyte transplant.

Repaired tissue was arthroscopically confirmed to harbor implant lesions, including cartilage tissue with good tissue integration with adjacent cartilage 12 months after surgery in one case. Indications for second-look arthroscopy included arthrofibrosis or meniscus lesions (Figure 6).

### Histologic Evaluation

No complications were observed in the repaired tissue for the other cases. HE and S-O staining revealed different characteristics of the osteochondral junction between normal and repaired cartilage. Histology of the biopsy specimens revealed repaired tissue, with cartilaginous tissue exhibiting positive S-O staining in one case. S-O staining provided the best distinction between red- or pink-stained sulfated glycosaminoglycans and green-stained subchondral bone. The detailed observation indicated that the superficial zone of repaired tissue contained predominantly spindle-shaped fibroblast-like cells. In contrast, most of the deeper repair matrix in all cases showed positive S-O staining. It had round-shaped cells in the cavity, suggestive of repaired tissue, which was particularly observed for the hyaline cartilage-like matrix.

**FIGURE 7:**
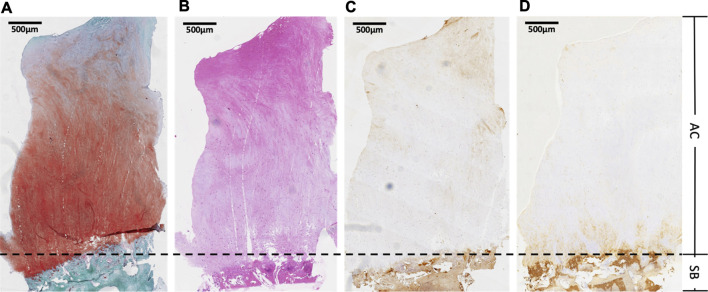
Histologic evaluation. The sample was taken from second-look arthroscopy performed 12 months after the operation. AC, articular cartilage; SB, subchondral bone **(A)** safranin O-fast green (S-O) stain for glycosaminoglycan distribution, **(B)** hematoxylin and eosin (HE) stain to observe general cell morphology,**(C)** immunohistochemically (IHC) with type-specific antibodies against collagen type I **(D)** immunohistochemically (IHC) with type-specific antibodies against collagen type II to evaluate collagen distribution of regenerated cartilage.

Furthermore, the interface between the repair cartilage and subchondral bone exhibited normal osteochondral junctions. A zonal structure in repaired cartilage was confirmed in one of the cases. As revealed via HE staining, the newly formed cartilage included naive chondrocytes with a round or oval shape, and the tidemark separated the cartilage. Subchondral bone cannot be clearly identified in freshly regenerated tissue. Given that mature chondrocytes look flatter relative to more basal zones in the superficial area and more spheroidal ones in the transitional or middle zone (Baumann et al., 2019), HE staining revealed oval or round-shaped cells with the phenotypic characteristics of native chondrocytes, which implied that the chondrocytes were still naive at this time. The S-O stain readily discriminated cartilage from the fast green-stained bone, indicating that the cells were surrounded by sulfated proteoglycan-rich ECM, typical of cartilage. Sections were immunostained for collagen type II and collagen type I, followed by observation via microscopy. Type II collagen deposition was observed on all type I scaffolds in addition to minimal staining for type I collagen, which suggested the generation of hyaline cartilage. Type II collagen was uniformly distributed at the deep zone of articular cartilage, while type I collagen was mainly deposited in the superficial cartilage layer (Figure 7).

## Discussion

This study demonstrated the efficacy of type I collagen-based MACT for cartilage regeneration in a prospective clinical study with a 2-year follow-up. Positive clinical and morphological outcomes were consistently observed in all 20 patients without any significant adverse events. Histological evidence confirmed the presence of regenerating hyaline-like cartilage in the knee with an articular cartilage lesion determined via biopsy in one case, and it also showed that repaired cartilage tended to emerge from the deep to the superficial layer. Further, quantitative T2 mapping indicated a difference between the regenerated cartilage and the surrounding hyaline cartilage.

In the current study, the KOOS was used as the primary outcome measure. All five KOOS domains improved equally in all patients, and this improvement was sustained throughout the 2-year follow-up period. Sports and recreation as well as quality of life scores were somewhat lower than those of the other categories, which might be due to fear-related psychological factors after the initial mechanical damage to cartilage. In other words, due to concerns of reinjury, patients felt hesitant to regularly participate in active exercise or other moderate physical activity recommended for rehabilitation. This suggested that appropriate psychological counseling had to be provided depending on the psychological characteristics of patients in order to promote recovery. Although the current study included fewer cases and follow-up times than these previous studies, the outcomes were still comparable. Moreover, direct comparisons between the present study’s findings and those of the previously mentioned studies might not be feasible owing to variations in patient demographics, follow-up duration, cell handling techniques, and histological grading systems. Nonetheless, the clinical outcomes of our study indicate considerable improvements following the operation. However, the higher baseline and shorter follow-up times in the present study might account for the relatively smaller improvement compared to previous literature.

The evidence also showed that the regenerated cartilage and subchondral bone were not fully integrated, and regenerated cartilage was still different from the surrounding hyaline cartilage. This finding was consistent with the results of quantitative MRI T2 mapping, as T2 values were still higher than those of the surrounding cartilage. In parallel, the arthroscopic examination of nascent tissue formation suggested that the immature cartilage tissue accounted for the regenerated cartilage presenting with generalized tissue fragility. Based on these observations, we speculate that the postoperative recovery phase was possibly more than 24 months. Although the current study demonstrated that MACI has limited short-term efficacy, it still had a promising clinical effect with many cartilage-like tissues filling the lesion, which was also reported in several previous studies. In a prospective clinical following 31 patients for 5 years after MACI, Ebert et al. (2017b) also reported favorable clinical outcomes. That is, all grafts were preserved on follow-up MRI, except for two graft failure cases due to complications. Moreover, Barie et al. (2020) aimed to establish whether MACI or second-generation ACI provided superior long-term outcomes based on patient satisfaction, clinical assessment, and MRI evaluation by following up 16 patients for an average of 9.6 years in a randomized clinical trial. Their findings suggested that the MACI method was equally effective for the treatment of isolated full-thickness articular cartilage lesions. Nevertheless, these studies came to a slightly different conclusion that the current operation did not achieve satisfactory results in patients during the short follow-up given the quantitative MRI T2 mapping. The reason for the different results reported from these studies was that the current study had a shorter follow-up time of 24 months, and the entire procedure was performed using open parapatellar mini-arthrotomy. Unlike our study, previous research transplanted implants in arthroscopic surgery with long-term follow-up, and they just adopt subjective evaluation methods without objective evaluation methods. Thus, the results were slightly different from ours. These findings suggest that open parapatellar mini-arthrotomy is not conducive to promote the repaired cartilage.

MRI has been widely performed for evaluating cartilage repair while avoiding the potential sampling bias associated with tissue biopsies, and MRI results confirmed that the lesion was filled with homogeneous tissue in addition to a high integration ratio (Welsch et al., 2009; Mumme et al., 2016). From general view, MRI would verify repaired tissue fill, maturation, and integration with the surrounding cartilage over time. It would seem that MOCART evaluation can help to extract more information from MRI, but it is still a subjective evaluation and lacks credibility. In probable, quantitative MRI is currently the best tool for assessing repair quality after implantation and before surgery. However, the utility of quantitative T2 mapping remains disputable because it could not detect the tissue microstructure characterization and cell phenotype. Nevertheless, it still provides a sufficiently detailed structural evaluation of regenerated cartilage without considering the somewhat limited resolution of currently available imaging technology (Salzmann et al., 2014). Our results for MRI T2 mapping could not detect morphological differences between the superficial and mid-to-deep zones of repaired tissue which could be confirmed via histologic elevation. Thus, although invasive, the histological assessment was likely the most reliable method for the detailed structural evaluation of cartilage repair.

Histological evaluation was performed on one of the 20 patients 12 months after the transplantation. Assessment of type II collagen distribution revealed hyaline-like cartilage in one patient who underwent second-look arthroscopy with biopsy. In the current work, one biopsy revealed a strong expression of collagen II in the deep layer cartilage, with a slight decrease of intensity in the superficial layer and middle part of the specimen, which illustrated the cartilage deep was hyaline cartilage. In the superficial layer, the repaired cartilage had been detected the accumulation of type I collagen with gradually decreasing toward the middle and deep, which implied the cartilage surface was still fibrocartilage. These showed that repaired cartilage tended to emerge from the deep layer to the superficial layer. Similarly, Biant et al. (2017) found similar corresponding results that hyaline cartilage or a mixture of hyaline–fibrocartilage were detected in 9 of 33 cases at an average of 15 months after MACI. We all have a short follow-up time, the follow-up time could be as short as only 1 year. However, Grevenstein et al. (2021) reported excellent histological results regarding the regeneration of hyaline articular cartilagen in all patients during a 16-month follow-up. This was a slightly different result from ours. Since he did not perform IHC studies for type I collagen, no subtle differences could be observed.

Several reports employed another technique for MACI, similar to the one we used. In a prospective study of ten patients undergoing arthroscopic gel-type autologous chondrocyte implantation (GACI), satisfactory clinical, radiological, and histological outcomes were reported, confirming the sufficient regeneration of hyaline-like cartilage, in parallel to the improved symptoms (Yoon et al., 2020). Since the GACI was infused in a liquid form, it was applied to the lesions regardless of their geometry, and the implant could then spread over the lesion site. Thus, GACI may represent a more convenient technique than other arthroscopic MACI methods. However, meticulous bleeding control was required for clear visualization of the lesion during the application of chondrocytes using a suction syringe and cotton bud. The amount of handling and manipulation necessary for implant preparation for an arthroscopic procedure might lead to additional chondrocyte apoptosis (Biant et al., 2017). Further, GACI lacked fixation, causing the graft to fall off, in addition to the issues of cell leakage and chondrocyte distribution. In contrast, as the implant in the current study was treated with a solid scaffold made of a non-flowing gel, it was fixed with fibrin glue and no membrane cover. MACT was performed as a mini-arthrotomy procedure for reimplantation. Furthermore, a small cadaveric study previously showed that 16 times as many viable cells remained on the membrane scaffold after implantation via open mini-arthrotomy compared to via arthroscopy (Biant et al., 2017).

The current study had several limitations. First, the procedure used herein was not compared to other operative techniques. Although performing reimplantation steps under mini-open arthrotomy and using the non-flowing gel scaffold might theoretically provide several advantages, further studies are required to compare operative time, postoperative rehabilitation, as well as clinical and radiologic outcomes following the arthroscopic delivery of autologous chondrocytes together with a type I collagen scaffold. Second, since a power calculation was not performed, the small sample size is an essential drawback of this study. Third, the follow-up time was relatively short for some evaluations. Clinical outcomes were previously followed for up to 5 years, and it was confirmed that improved clinical scores were maintained for an extended period (Ebert et al., 2011). Finally, histological assessments were conducted on a single patient 12 months after implantation, with relatively limited histological evaluation. Therefore, the current study could not assess implant integrity nor any change in the composition and distribution of type II collagen in the long term. This posed a significant challenge for obtaining comprehensive data to support our conclusion.

The type I collagen-based MACT for treating chondral or osteochondral lesions of the knee is clinically effective, yielding significant improvement in functionality and pain levels. It presents immense potential for regenerate cartilage, and the finds and limitations of this study will direct our near-term future research.

## Data Availability

The original contributions presented in the study are included in the article/Supplementary Material, further inquiries can be directed to the corresponding authors.

## Abbreviations

AC, Articular cartilage; ACI, autologous chondrocyte implantation; GACI, gel-type autologous chondrocyte implantation; HE, hematoxylin and eosin; ICRS, International Cartilage Repair Society; IHC, immunohistochemistry; KOOS, knee injury and osteoarthritis outcome score; MACI, matrix-associated autologous chondrocyte implantation; MACT, matrix-assisted autologous chondrocyte transplantation; MOCART, magnetic resonance observation of cartilage repair tissue; MRI, magnetic resonance imaging; PDW, proton density-weighted; ROI, the region of interest; SB, subchondral bone; S-O, Safranin O-fast green; SPAIR, spectral attenuated inversion recovery.
